# Analyzing the Influencing Factors and Workload Variation of Takeover Behavior in Semi-Autonomous Vehicles

**DOI:** 10.3390/ijerph19031834

**Published:** 2022-02-06

**Authors:** Hui Zhang, Yijun Zhang, Yiying Xiao, Chaozhong Wu

**Affiliations:** 1 Intelligent Transportation Systems Research Center, Wuhan University of Technology, Wuhan 430063, China; zhanghuiits@whut.edu.cn (H.Z.); zyjits@whut.edu.cn (Y.Z.); shadowxyy@outlook.com (Y.X.); 2 National Engineering Research Center for Water Transport Safety, Wuhan 430063, China

**Keywords:** shared driving, workload, influencing factors, NASA-TLX scale

## Abstract

There are many factors that will influence the workload of drivers during autonomous driving. To examine the correlation between different factors and the workload of drivers, the influence of different factors on the workload variations is investigated from subjective and objective viewpoints. Thirty-seven drivers were recruited to participant the semi-autonomous driving experiments, and the drivers were required to complete different NDRTs (Non-Driving-Related Tasks): mistake finding, chatting, texting, and monitoring when the vehicle is in autonomous mode. Then, we introduced collision warning to signal there is risk ahead, and the warning signal was triggered at different TB (Time Budget)s before the risk, at which time the driver had to take over the driving task. During driving, the NASA-TLX-scale data were obtained to analyze the variation of the driver’s subjective workload. The driver’s pupil-diameter data acquired by the eye tracker from 100 s before the TOR (Take-Over Request) to 19 s after the takeover were obtained as well. The sliding time window was set to process the pupil-diameter data, and the 119-s normalized average pupil-diameter data under different NDRTs were fitted and modeled to analyze the variation of the driver’s objective workload. The results show that the total subjective workload score under the influence of different factors is as follows: obstacle-avoidance scene > lane-keeping scene; TB = 7 s and TB = 3 s have no significant difference; and mistake finding > chatting > texting > monitoring. The results of pupil-diameter data under different factors are as follows: obstacle-avoidance scene > lane-keeping scene; TB = 7 s > TB = 3 s; and monitoring type (chatting and monitoring) > texting type (mistake finding and texting). The research results can provide a reference for takeover safety prediction modeling based on workload.

## 1. Introduction

With the development of advanced driving assistance systems, the vehicle is becoming increasingly intelligent, and autonomous driving has become a research hotspot in the field of transportation in recent years [1]. For drivers, it can release them from the main driving task, and they can perform different NDRTs [2], thus improving the comfort of driving. In 2014, SAE (the Society of Automotive Engineers) proposed a classification standard for autonomous vehicles. In September 2016, it updated the standard to classify vehicles into six levels based on intelligence degree, ranging from level 0 to level 5. They are driver, assisted, partially automated, highly automated, fully automated, and autonomous, respectively [3]. Although the intelligence of vehicles has been greatly developed, due to the limitations of automation technology, laws, and regulations, it is difficult to realize autonomous driving in the short term. For a long time in the future, autonomous vehicles will still be driver controlled, and most autonomous vehicles will be “highly automated” (Level 3) [4]. In level 3, the autonomous driving system still needs the driver to take over the vehicle temporarily, for example, when the driving environment cannot be properly recognized, the autonomous driving system suddenly fails, the current driving environment is not suitable for autonomous driving, etc. When the above situation occurs, the vehicle will send out a takeover request to the driver, and the control of the vehicle will be transferred from autonomous driving to manual driving. The driver needs to focus on the main driving task rather than NDRTs and take over the vehicle immediately. Drivers who take over the vehicle in above situations are passive takeover type [5] drivers who need to deal with more information than that in a traditional driving environment. When drivers are not ready, passive takeover will affect a driver’s cognitive process, and drivers cannot safely take over the vehicle, potentially leading to a serious traffic accident.

Previous studies have proven that the takeover process and performance evaluation are the most basic and significant research part of level 3 autonomous driving [6]. Takeover performance is affected by many factors, including driver’s cognitive process, whether drivers perform NDRTs [7], types of NDRTs [8], complexity of driving scene [9], weather conditions [10], traffic density [11], methods [12] and intensity [13] of takeover requests, urgency [14] of takeover requests, duration of automatic driving [15], vehicle type [16] and HMI (Human Machine Interface) design [17], familiarity with the driving system, driver’s age [18], gender [19], mood [20], vehicle failures [21], etc.

However, with the improvement of automation, drivers have more disposal time [22], making it more possible for drivers to perform NDRTs (Non-Driving-Related Task) [23]. Sometimes, drivers may perform more than one NDRT [24], such as playing on a mobile phone while talking. These NDRTs could occupy a driver’s visual, auditory, and cognitive resources at certain degrees. According to the limited resources theory summarized by psychologist Miller, resources that could be used for an individual are limited, and performing a task will occupy certain resources. When the NDRTs occupy too many resources, each type of resources allocated to the driving task could be insufficient, and drivers could be cognitively overloaded, resulting in the decrease of driver attention and alertness and a negative impact on driving safety. ZeebK [25] found that when takeover request is merely urgent, there is no significant difference between whether drivers are performing NDRTs and takeover reaction time, but it could affect the quality of a driver’s takeover, such as the average lane deviation of drivers reading newspapers and watching videos is 8–9 cm more than drivers who do not perform NDRTs. Therefore, it is essential to study the impact of NDRTs on takeover performance.

To make the experiment safe, repeatable, and controllable, most experiments studying on workload variation of takeover behaviors are carried out in driving simulators [26]. Nowadays, rare researches focus on the workload changes in field experiment and simulated experiment, respectively, and they show contradictory results [27,28].

To conclude, this study is based on the shared driving simulated experiment and uses NASA-TLX scale and Dikablis Glasses eye tracker to obtain subjective and objective workload data of drivers. The influencing factors of subjective and objective workload in the process of takeover during autonomous driving are investigated to reveal the variation of workload before and after the driver performs NDRTs. In addition, the effects mechanism of integration of different factors on workload and takeover performance are explored. This study could provide the basis for autonomous-driving vehicle warning system and urban road safety.

## 2. Materials and Methods

### 2.1. Participants

According to previous study [26], the current study recruited 37 drivers (12 females) with skilled driving experience (participants) to conduct the experiment. The specific requirements are: (1) the drivers are close in age, holding a driving license, and familiar with traffic laws and regulations; (2) good health and have no history of major diseases in the recent 5 years; (3) normal work and rest and no alcohol, drugs, and other bad habits; (4) no 3D vertigo and good adaptability to the simulator; and (5) the glasses have no high flash and eyes that have not undergone operation; the demographic characteristics of the participants are shown in Table 1.

### 2.2. Experimental Equipment and Questionnaire

1. Simulated Driving Experiment Platform

**Figure 1:**
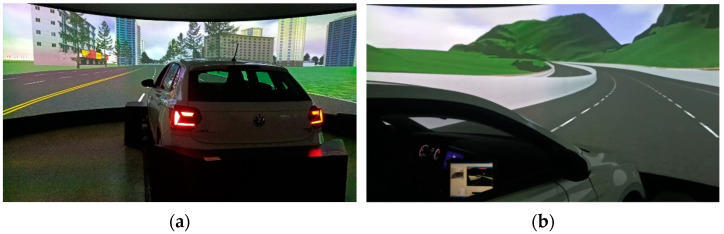
Simulated driving experiment platform. (**a**) Simulator modified from a real car; (**b**) Screen displayed.

In this experiment, UC-WIN /Road13.0 software (FORUM8, Tokyo, Japan) was used to design a simulated experiment driving scene, and the simulator was modified from a Shanghai Volkswagen POLO car, shown in Figure 1. UC-win /Road13.0 software controlled 7 computers, among which 2 computers generated the simulated scenes of left and right rearview mirrors and sent them to the small LCD screens acting as rearview mirrors on both sides of the simulator. Five computers generated simulated road scenes, and scenes were delivered to five large display screens in front of the simulator to form a simulated driving environment.

2. NASA-TLX scale

The NASA-TLX scale was used to evaluate the subjective workload in this experiment, which contained six indicators: mental demand, physical demand, time demand, satisfaction, effort level, and frustration level. The 6 indicators of the NASA-TLX scale are divided into 10 grades according to requirements or degrees, and they are ranked from the first to the sixth according to the degree of correlation to the total load, and thus, the final total subjective workload score was calculated. NASA-TLX scale is shown in the attachment.

3. Dikablis Glasses Eye Tracker

**Figure 2:**
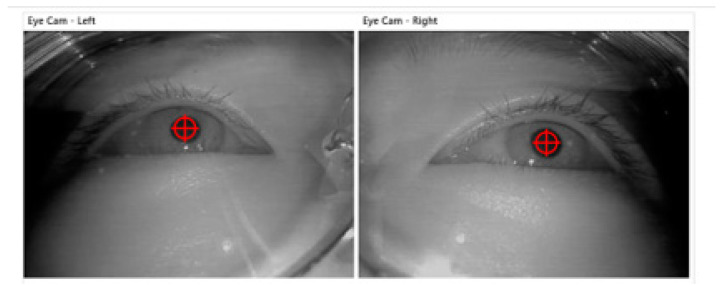
Pupil calibration using Dikablis Glasses eye tracker.

The eye tracker is widely used to measure workers’ situation awareness from objective aspect [29]. Dikablis Glasses eye tracker is a non-invasive eye tracking system consisting of three channel cameras. The first and second cameras for visual characteristics are responsible for collecting eye movement data of left and right eyes, shown in Figure 2; the third camera is responsible for collecting scene information in front of drivers. It can collect the driver’s line of sight, area of interest, PERCLOS, gaze, saccade, pupil, and other data.

### 2.3. Experimental Procedure

**Figure 3:**
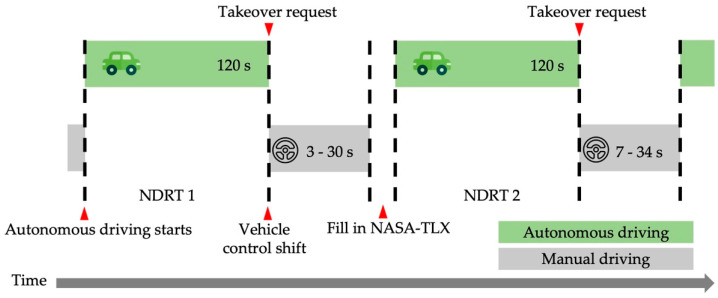
Takeover procedure.

Sleep quality and different driving time could affect the driver’s vehicle maneuverability. Therefore, the participants were required to sleep more than 7 h the day before the experiment, and the experiment lasted from 9 a.m. to 12 a.m. in the morning. Before the experiment, the instructor confirmed participants’ physical condition, sleep status, and driving experience. Then the whole process of the experiment was then introduced. Each participant signed an informed consent form, a data-use agreement, and a personal information registration form. Then, after the participants put on the Dikablis Glasses eye tracker, they could enter the simulator after the adjusting of the eye tracker. After the participants entered the pilot simulator, they first became familiar with the manual driving and autonomous-driving takeover methods. Then, the participants were informed of the NDRTs that needed to be performed during the autonomous driving. The whole takeover procedure of NDRT1 and NDRT2 is shown in Figure 3. After the experiment began, the vehicle would initially maintain its autonomous driving at the speed of 90 km/h for 2 min. When the vehicle reached the takeover point in the simulated experiment scene, a takeover request sound was sent to the driver, and the vehicle was transferred from autonomous driving to manual driving. At this point, the participant had to take over the vehicle. After the vehicle was stably taken over by participant, the vehicle needed to be returned to the middle lane as much as possible, and the speed had be maintained between 80–120 km/h. After manual driving for some time, the participant saw the stop sign on the right roadside. After driving past the stop sign, the participant parked the vehicle at the emergency lane, at which point a full takeover was completed. There were 16 takeovers in the whole shared-control driving simulation experiment, and the NASA-TLX scale was filled out after each takeover. Table 2 shows the sequence of 16 NDRTs and their TB and takeover scenes. The eye-related data in the whole experiment process were collected by the (Ergoneers GmbH, Egling, Germany).

### 2.4. Data Analysis

1. NASA-TLX Scale Data

A total of 560 NASA-TLX valid questionnaires from 35 participants were obtained after selection. Next, we input the questionnaire data filled out by the participants into the SPSS software and calculated the weight of each indicator according to the ranking of the contribution to the total load and finally calculated the total subjective workload score based on the weight and the scores of 6 dimensions, calculated as follows:

(1) $$S=\frac{1}{15}\overset{6}{\underset{i=1}{\sum }}X_{i}W_{i}$$

where *S* is the total score of NASA-TLX subjective workload, *X_i_* is the score of the *i* dimension, and *W_i_* is the weight of the *i* dimension.

To obtain the consistency or reliability of the questionnaire data, SPSS software was used to analyze the reliability of the NASA-TLX questionnaire, and the Cronbach’s alpha was 0.763, indicating that the questionnaire has good reliability and consistency. KMO (Kaiser–Meyer–Olkin) and Bartlett sphere test were performed on the NASA-TLX questionnaire; the KMO value was 0.854, and the Sig. value of Bartlett sphere test was 0.00, which is far less than 0.01, reaching the significance level and indicating that the questionnaire data were suitable for factor analysis.

Before performing variance analysis and correlation analysis, it was necessary to check whether the original data obeyed a normal distribution or an approximate normal distribution. The original data sample size used in this study is less than 5000, so it is more suitable to choose the non-parametric test method, such as Shapiro–Wilk test, which is suitable for small sample data. Under the test level of α = 0.05, the W test result of the subjective workload total score is 0.04, *p* = 0.04 < 0.05, and the sample data of the subjective workload total score do not follow a normal distribution. The W test result of the distraction degree score is 0.11, *p* = 0 < 0.05, and the sample data of the distraction degree score also do not obey the normal distribution.

**Figure 4:**
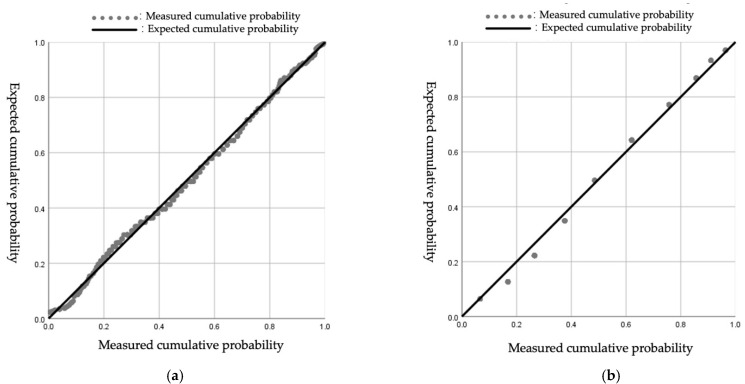
The normal P-P diagram of subjective workload total score and distraction degree score. (**a**) The normal P-P diagram of subjective workload total score; (**b**) The normal P-P diagram of distraction degree score.

When using the non-parametric test method, it will be affected by the data sample size. It is necessary to combine histograms, P-P diagrams, and other methods to make a comprehensive judgment to determine whether the original data obey the normal distribution. The normal P-P diagram of subjective workload total score and the distraction degree score are as follows: it can be seen from Figure 4 that the data points mostly overlap with the theoretical diagonal, indicating that the data are considered to obey a normal distribution.

Due to the different results of the previous two methods, the descriptive statistical function in SPSS was used to make a basic statistical description of the subjective workload total score and distraction degree score. The detailed description statistics are shown in Table 3.

The skewness value of the subjective workload total score data distribution is 0.17 (0.11), the skewness Z-score = 0.17/0.11 = 1.55, the kurtosis value −0.03 (0.23), and the kurtosis Z-score = −0.03/0.23 = −0.13. The skewness value is approximately equal to 0, and the kurtosis value is also approximately equal to 0. Both the skewness Z-score and the kurtosis Z-score are in the range of (−1.96, +1.96), so the total score of subjective workload is considered to obey the normal distribution.

The skewness value of the distraction degree score data distribution is 0.14 (0.10), the skewness Z-score = 0.14/0.10 = 1.41, the kurtosis value is −0.93 (0.20), and the kurtosis Z-score = −0.93/0.20 = −4.58. The skewness value is approximately equal to 0, the kurtosis value is approximately equal to 1, and the skewness Z-score is in the range of (−1.96, +1.96), but the kurtosis Z-score is not, so the distraction degree score data do not follow a normal distribution.

Comprehensively judging from three normal distribution test methods, it is believed that the subjective workload total score obeys the normal distribution or the approximate normal distribution, and the original data of the distraction degree score do not obey the normal distribution or the approximate normal distribution.

2. Eye Tracker Data

**Figure 5:**
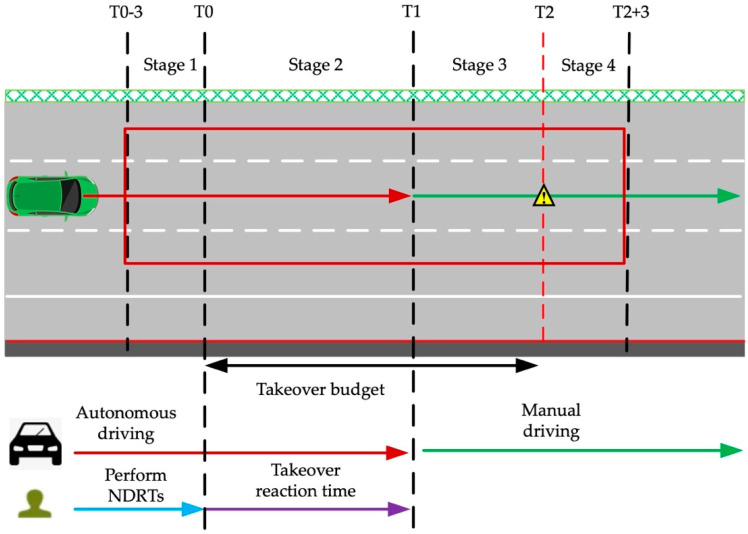
Different stages in a takeover.

Existing research shows that [30] when workload or psychological pressure increases, the pupils can expand involuntarily. When workload or psychological pressure decreases, the pupils can return to normal state. Since the shared driving simulated experiment is carried out indoors under the same light, the influence of light on pupil area can be ignored. Studies have shown that when drivers are tired, it can also affect the size of their pupils. In conclusion, the change of pupil diameter can reflect the change of driver’s workload. Therefore, the pupil diameter was selected as the characteristic indicator to measure driver’s objective workload. When the pupil diameter is larger, it indicates that the driver’s workload is higher. In this study, pupil-diameter data are divided into four stages, and the range and description of the four stages are shown in Figure 5 and Table 4.

When the driver blinks, the eye tracker cannot recognize the complete pupil, which will cause the pupil diameter at the corresponding time to be 0. Therefore, the pupil-diameter data at the time of blinking needs to be deleted. We replaced the value of 0 in the original data with a moving median of window length 5 (including the left and right 5 data points). In this study, the 3σ principle was used to eliminate outliers. However, the data should obey normal distribution or approximate normal distribution.

It had been verified that the skewness coefficient and kurtosis coefficient of pupil-diameter data are both less than 1, and the P-P graph obeyed the normal standard. The data are considered to be approximately normal distribution, and the 3σ criterion was adopted to eliminate the outliers. In order to eliminate the influence of the individual difference in the original pupil diameter of drivers on the experimental data, the data were normalized by Min-Max standard, and the function is as follows:

(2) $$P_{j}^{*}=\frac{P_{j}-P_{jmin}}{P_{jmax}-P_{jmin}}$$

where $P_{j}^{*}$ is the pupil diameter of the participant j after normalization, *P_j_* is the pupil diameter of the participant j before normalization, *P_jmax_* is the maximum value of pupil diameter, and *P_jmin_* is the minimum value of pupil diameter.

In this study, the change of pupil diameter was analyzed by means of normalized mean and standard deviation of pupil diameter. The mean pupil diameter *P_mean_* and standard deviation *P_std_* from stage 1 to 4 are calculated as follows:

(3) $$P_{mean}=\frac{1}{N}\overset{N}{\underset{i=1}{\sum }}P_{i}^{*}$$

(4) $$P_{std}=\sqrt{\frac{1}{N}\overset{N}{\underset{i=1}{\sum }}{\left(P_{i}^{*}-P_{mean}\right)}^{2}}$$

where *N* is the number of sampling points in each stage of the pupil area data, and $P_{i}^{*}$ is the pupil area corresponding to the sampling point *i*.

**Figure 6:**
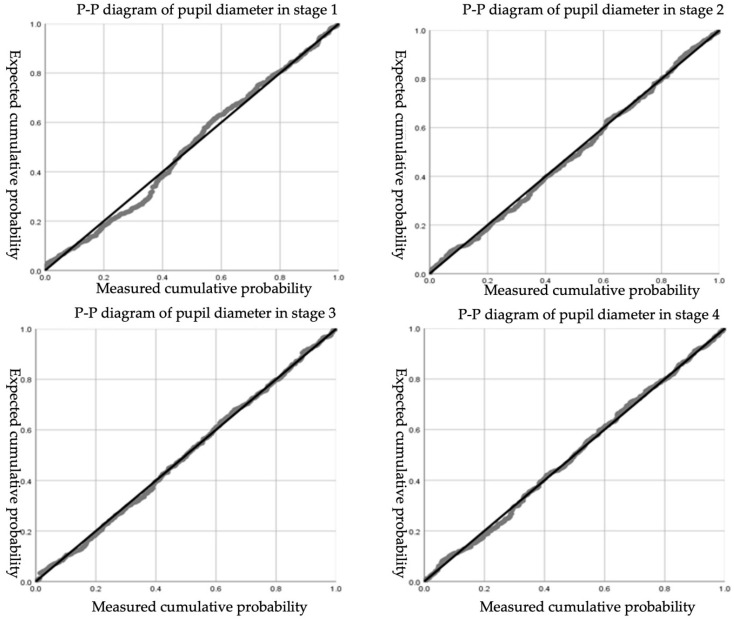
P-P diagram of normalized pupil diameter for different stages.

Normalized pupil diameter of all participants in the four stages is tested for normal distribution, and the P-P diagram and the results of the normality test are shown in Figure 6 and Table 5. The data in the four stages are all normally distributed.

## 3. Results

### 3.1. Influencing Factors Analysis of Workload during Takeover

#### 3.1.1. Influencing Factors Analysis of Subjective Workload

Spearman correlation analysis and Kendall rank-correlation analysis are used to explore different takeover scenes, TB distraction degree scores, and the subjective workload total score in all NDRTs situations. The results are shown in Table 6.

The subjective workload total score is significantly correlated with the difficulty of the takeover scene (*p* < 0.01), indicating that the participant’s subjective workload is higher when the takeover scene is an obstacle-avoidance scene. There is also a significant correlation with TB (*p* = 0.049 < 0.05) and a significant negative correlation with NDRT (*p* < 0.01), indicating that drivers who perform text-based tasks have a higher subjective workload when they take over than when they perform monitoring tasks. There is a significant positive correlation between the subjective workload total score and the subjective distraction degree score (*p* < 0.01), and the correlation coefficient is 0.544, indicating that there is a moderate linear correlation between them. When performing NDRT with a greater distraction degree, the higher the driver’s subjective workload total score will be.

#### 3.1.2. Influencing Factors Analysis of Objective Workload

Kendall rank-correlation analysis is used to explore the correlation between different time segments and different takeover scenes, TB, and NDRT types. The results are shown in Table 7.

It can be found that TB is significantly correlated with pupil diameter (*p* < 0.01) at stage 3 and stage 4. This indicates that the higher the TB is, the more time drivers spend observing the driving environment to ensure the safety of takeover, and the workload is higher. In the stages 2–4, there is a significant correlation with NDRT (*p* < 0.01), indicating that different tasks have an impact on the workload occupied by the driver when taking over the vehicle again.

### 3.2. Variation of Workload during Takeover

#### 3.2.1. Variation of Subjective Workload of Drivers

1. Subjective workload variations under the independent influence of single factor

The total score of the driver’s subjective workload is influenced by factors, such as takeover scenes, TB, NDRT, etc. To further explore the impact of different levels of each factor on workload, statistical methods were used to explore the differences between groups in the driver’s subjective workload total score.

The Mann–Whitney U [9] test was used to determine whether the distribution of two groups of independent samples from the two total samples is significantly different. The Kruskal–Wallis test is a rank-sum test, which is an upgraded method of the Mann–Whitney U test in the case of multiple independent samples. Its null hypothesis H is that the distribution of multiple independent samples from multiple total samples is not significantly different. First, the multiple sets of sample data are mixed and sorted in ascending order to calculate the rank of each variable value. Second, whether there is a significant difference in the mean of the rank of each group is judged. If the values are merely different, it is considered that there is no significant difference in the distribution of multiple total samples. Otherwise, it is considered that there are significant differences in the distribution of multiple total samples, which is calculated as follows:

(5) $$\mathrm{KW}=\frac{Within\;Groups\;Sum\;of\;Squares}{Rank\;Variance\;of\;Total\;Samples}=\frac{12}{n\left(n+1\right)}\overset{K}{\underset{i=1}{\sum }}n_{i}{\left(\frac{R_{i}}{n_{i}}-\frac{n+1}{2}\right)}^{2}\;=\frac{12}{n\left(n+1\right)}\overset{K}{\underset{i=1}{\sum }}\frac{R_{i}{}^{2}}{n_{i}}-3\left(n+1\right)$$

where *R_i_* is the rank sum of each group, and *n_i_* is the number of samples in each group.

If there is a knot value (the amount of data with the same rank value) in the sample, the correction coefficient C is:

(6) $$C=1-\frac{\sum \left(\tau_{i}{}^{3}-\tau_{i}\right)}{n^{3}-n}$$

where $\tau_{i}$ is the number of j knot value.

The adjusted Kruskal–Wallis calculation formula is as follows:

(7) $$KW_{c}=KW/C$$

- Impact of different takeover scenes on subjective workload

The MW test was used to analyze the subjective workload total scores under different takeover scenes. From Table 8, there are significant differences in the subjective workload total scores for different takeover scenes. According to statistics, the driver believes that vehicle maneuvering behavior when it is necessary to take over in time to avoid an accident consumes more cognitive resources than in the case of unnecessary takeover.

- Impact of different TB on subjective workload

The MW test was used to analyze the subjective workload total scores under different takeover time budgets. From Table 9, for different takeover time budgets, there is no significant difference in the driver’s subjective workload total score. According to statistics, different takeover time budgets have little effect on the driver’s subjective workload.

- Impact of different NDRTs on subjective workload

KW test was used to analyze the subjective workload total score under different NDRTs, as shown in Table 10. For different NDRT, the driver’s subjective workload total score is significantly different. Comparing the task of “mistake finding” with the task of “texting”, “chatting”, and “monitoring”, respectively, it can be concluded that the driver’s subjective workload will increase when performing relatively more difficult tasks. Based on all the data of the participants, non-parameters tests were carried out on the different NDRTs in pairs, and there is no significant difference among the “mistake finding” task, “texting” task, and “chatting” task. The subjective workload total score of the “monitoring” task and other tasks is significantly different (*p* < 0.01).

2. Variation of subjective workload under the interactive influence between factors

Taking the interaction between the two related factors into consideration, the non-parametric test method was used to analyze the data of different groups in pairs. The influence of different factors on the subjective workload score was analyzed.

- Impact of different TB*different NDRTs on subjective workload

**Figure 7:**
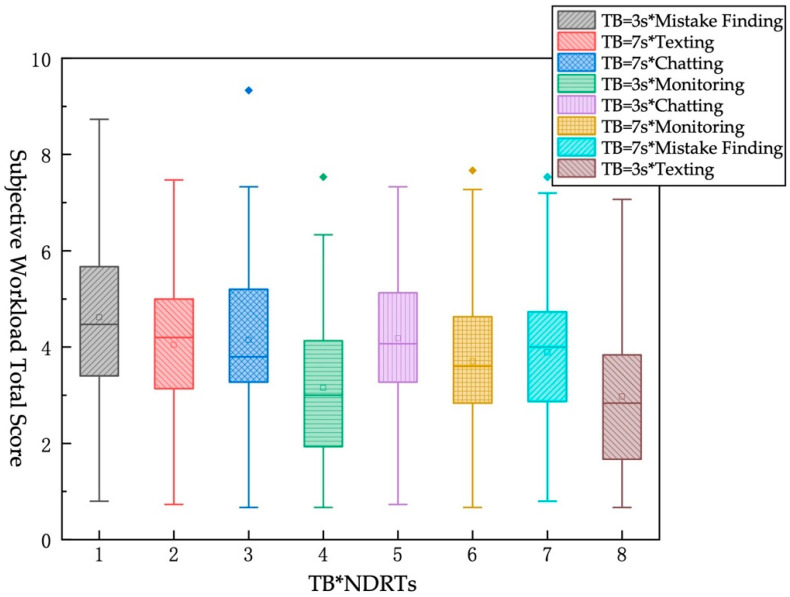
KW test chart of subjective workload total score under different TB*different NDRT.

KW test was used to analyze the subjective workload total score of different TB and different NDRT. From the Table 11 and Figure 7, it indicated that the longer the TB, the lower the subjective workload total score under the same NDRT. The pairwise difference analysis of the pairs found that when TB = 3 s, the subjective workload total score of monitoring task is significantly different from the other three tasks, and there is no significant difference among other tasks. When TB = 7 s, there is a significant difference between the monitoring task and the “mistake finding” task and the “chatting” task but shows no significant difference from the texting task.

- Impact of different TB*different Takeover Scene on subjective workload

**Figure 8:**
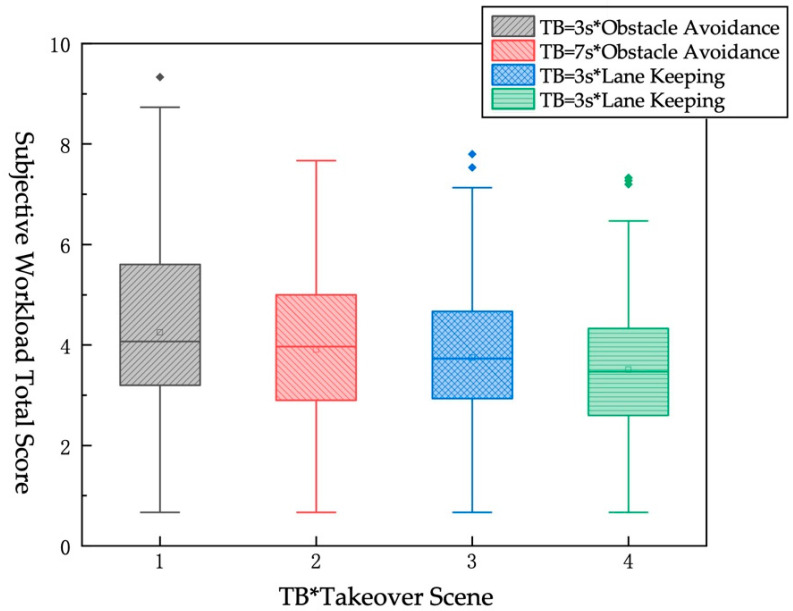
KW test chart of subjective workload total score under different TB*different Takeover scene.

The KW test was used to analyze the subjective workload total score for different TBs and different takeover scenes. From Table 12 and Figure 8, in the same takeover scene, the less TB for takeover, the higher the driver’s workload. Under the same TB, the greater the risk of scene, the higher the driver’s workload. The pairwise difference analysis of the pairs found that only pair 1 and pair 4 have significant differences.

- Impact of different NDRTs*different Takeover Scene on subjective workload

**Figure 9:**
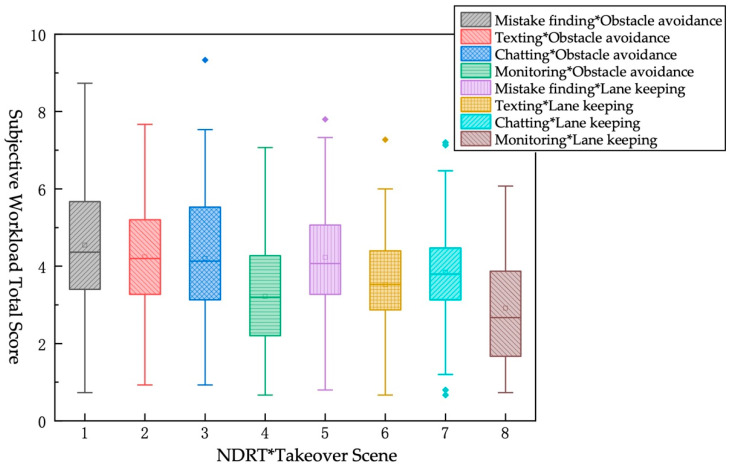
KW test chart of subjective workload total score under different NDRT*different Takeover scene.

The KW test was used to analyze the subjective workload total scores under different NDRTs and different takeover scenes. From Table 13 and Figure 9, the lower the risk of takeover scenes, the lower the subjective workload total score under the same NDRT. Further analysis found that in the obstacle-avoidance scene, the subjective workload total score under the monitoring task is significantly different from the other three tasks, and there is no significant difference among other tasks. In the lane-keeping scene, the monitoring task is significantly different from the “mistake finding” task and the “chatting: task, but there is no significant difference from the “texting” task.

#### 3.2.2. Variation of Objective Workload of Drivers

In this study, pupil diameter was selected as the objective workload variation parameter, and the significance impact analysis method mentioned was used to consider the influence of different factors and levels on multiple stages before and after takeover so as to explore the variations of objective workload.

1. Objective workload variations under the independent influence of single factor

Drivers’ workload is affected by takeover scene, TB, NDRT and other factors. To further explore the influence of different levels of each factor on workload, this study used statistical methods to explore the differences between groups of drivers’ workload.

- Impact of different takeover scenes on objective workload

Two takeover scenes were designed for shared driving. In the obstacle-avoidance scene, the driver needs to control the vehicle to get across the faulty vehicle in front after receiving the takeover request. In the lane-keeping scene, the vehicle keeps driving in the middle of the lane when the lane line disappears. The normalized pupil-diameter data of drivers in different takeover scenes were extracted, and the mean value of normalized pupil diameter in obstacle-avoidance scene and lane-keeping scene was calculated, as shown in Table 14.

**Figure 10:**
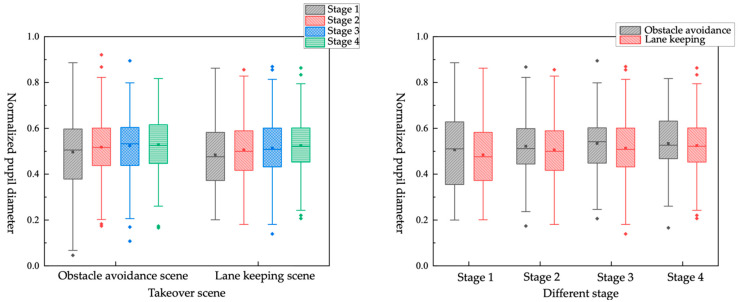
Box plot of normalized pupil diameter in different takeover scene and stages.

Figure 10 shows that in different takeover scenes, the normalized pupil diameter of the driver increases before and after the takeover request (from stage 1 to stage 2). In addition, before and after the vehicle takeover (from stage 2 to stage 3), the normalized pupil diameter of the driver also increases, and the normalized pupil diameter increases slightly when vehicle is near the faulty scene (from stage 3 to stage 4). It shows that although the driver has taken over the vehicle, it will take some time for the driver to fully recover his cognitive ability. This finding is consistent with the conclusion of Merat et al. [31]. Merat found that drivers can recover their operating ability within 1 to 2 s, but it takes longer to recover cognitive ability.

When the takeover scene is the obstacle-avoidance scene, the normalized pupil diameter of the driver is larger than that of the lane-keeping scene in the four stages, respectively. It indicates that under the circumstance when the drivers must take over the vehicle to ensure safety, the cognitive load occupied could be higher.

- Impact of different TB on objective workload

This experiment designed two takeover time budgets, 3 s and 7 s, respectively. We extracted the normalized pupil-diameter data of the driver under different TBs and calculated the mean value of the normalized pupil diameter when TB was 3 s and TB is 7 s; the data are shown in Table 15.

**Figure 11:**
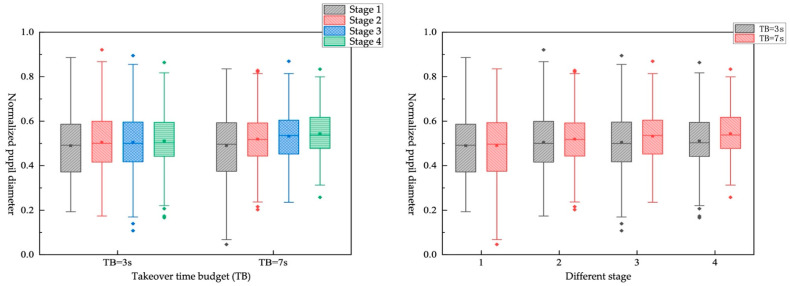
Box plot of normalized pupil diameter in different TB and stages.

It can be shown in Figure 11 when the TB is 3 s, the normalized pupil diameter of the driver changes merely in the stage 3 and 4. It can be obtained that when it is an urgent takeover, the driver cannot sufficiently process the driving environment information before the takeover, and the cognitive ability is not recovered in a short time. When the TB is 7 s, the driver will have a larger normalized pupil diameter, and the change is greater than when the TB is 3 s. It shows that when the driver has enough time to take over the vehicle, in order to take over the vehicle more safely, the brain can provide enough cognitive resources for processing driving environment information.

- Impact of different NDRT on objective workload

**Figure 12:**
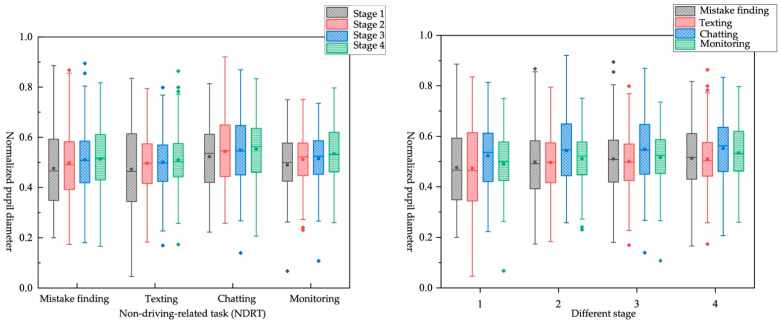
Box plot of normalized pupil diameter in different NDRTs and stages.

Four NDRTs are designed in this experiment, namely “mistake finding”, “texting”, “chatting”, and “monitoring”. We extracted and calculated the average value of the normalized pupil diameter of the driver under different NDRTs, as shown in Table 16. As shown in Figure 12, when performing different NDRTs, the normalized pupil diameter of the driver increases from stage 1 to stage 4. Consistent with the previous conclusions, it takes some time for drivers to fully recover their cognitive abilities.

In this study, the Kruskal–Wallis test was used to analyze the influence of different NDRTs on the pupil area at each stage. From 3 s before the takeover request to 3 s after the faulty scene, the normalized pupil diameter in the obstacle-avoidance scene is larger than that in the lane-keeping scene, indicating that in the obstacle-avoidance scene where the vehicle must be taken over in time, the driver needs to consume more cognitive resources. When TB is 7 s, the driver has enough time to take over the vehicle. To take over the vehicle more safely, the brain can provide sufficient cognitive resources for the processing of driving environment information. When the task is “monitoring”, the driver only focuses on the road conditions ahead without manual operation, and he will have a higher cognitive load. When the task is “chatting”, the brain needs to analyze the information from the current driving environment and needs to answer the questions of the instructor, which occupies more cognitive resources. When the driver is immersed in the “texting” NDRT, he only needs to think about the text content and perform manual input operations. Relatively speaking, “monitoring” occupies more cognitive load than “texting”.

2. Variation of objective workload under the interactive influence between factors

We considered the interaction between the two related factors, analyzed the data in pairs, and found the correlation degree of different factors on the driver’s normalized pupil diameter.

- Impact of different TB*different NDRTs on objective workload

**Figure 13:**
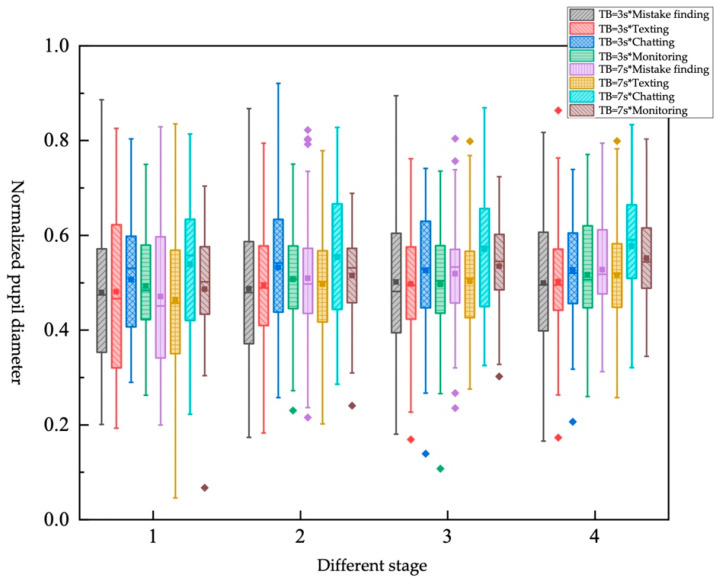
Box plot of normalized pupil diameter under different TB*different NDRTs.

There are eight combinations from TB and NDRTs shown in Table 17. The box diagram is shown in Figure 13. It can be found that different TBs have little effect on the “texting” tasks in stage 1 but have an obvious impact on the “monitoring” tasks. That is, when the TB is larger, the vehicle is far from the faulty scene, and the driver needs to continue paying attention to obtain actual faulty scene information, and it will occupy more cognitive resources. Different TBs have a greater impact on the “texting” tasks in stage 1, stage 2, and stage 3. The driver cannot obtain real-time driving environment information due to participation in the “texting” task. The larger the TB, the more time it gives the driver to obtain information. However, “monitoring” tasks involve continuously acquiring driving environment information in real time before the takeover request is sent out, and different TBs have little impact on them. The effect of different NDRTs on the driver’s normalized pupil diameter under different TB is consistent with the conclusions of the single-factor study.

- Impact of different TB*different takeover scene on objective workload

**Figure 14:**
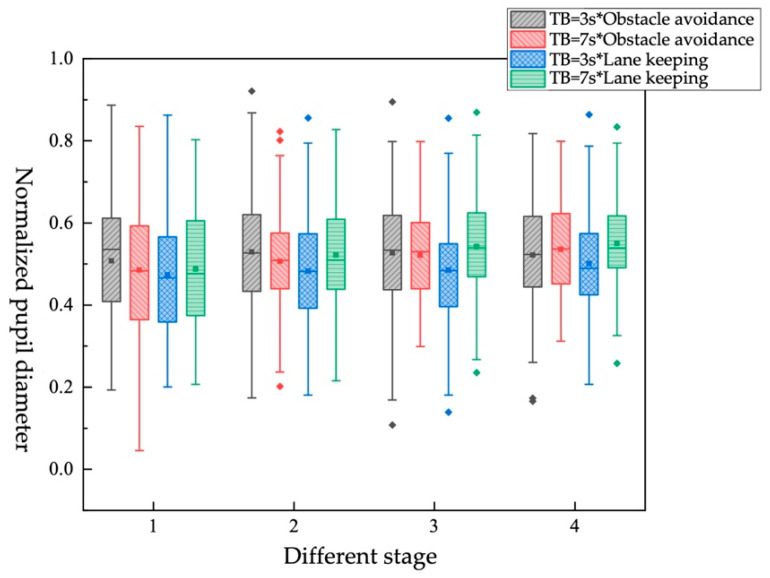
Box plot of normalized pupil diameter under different TB*different takeover scene.

Four pairs were generated by combining TB and takeover scene, as shown in Table 18 and Figure 14. It can be found that in the obstacle-avoidance scene, when the TB is 3 s, the driver’s pupil diameter changes from stage 1 to stage 2 is less than when the TB is 7 s. In stage 3, the pupil diameters in the two cases gradually become the same. In the lane-keeping scene, in the four stages, the pupil diameter of the driver when TB is 3 s is smaller than when TB is 7 s. According to the ranking obtained by single-factor analysis (obstacle-avoidance scene > lane-keeping scene, TB = 7 s > TB = 3 s), it can be obtained that TB has a higher impact on pupil diameter than the takeover scene.

- Impact of different NDRTs*different takeover scene on objective workload

**Figure 15:**
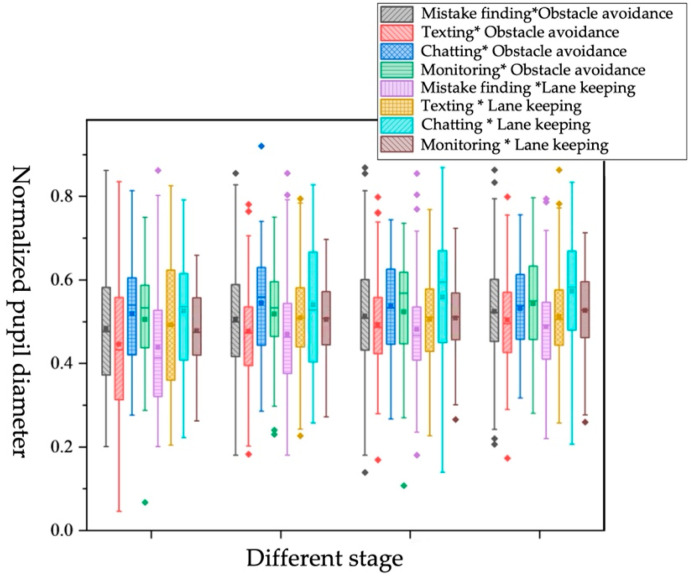
Box plot of normalized pupil diameter under different NDRTs*different takeover scene.

Eight pairs were generated by combining NDRTs and takeover scene, as shown in Table 19 and Figure 15. In all NDRTs*takeover scene pairs except for chatting*obstacle avoidance, the normalized pupil diameter of the driver increased from stage 1 to stage 4. The pupil diameter under the chatting*obstacle avoidance pair increased from stage 1 to stage 2, followed by a slow decrease trend, while the chatting*lane keeping pair increased in all stage. It can be explained that the driver performs a chatting task that occupies more cognitive resources before taking over the vehicle, and when the takeover scene is obstacle avoidance, the driver obtains information about the faulty vehicle in front before the takeover request, and after changing lanes safely with cautious operations, drivers will gradually relax. When the takeover scene is lane keeping, the driver needs to pay attention to the driving environment information to keep the vehicle in the middle lane and still maintain a high level of alertness. For monitoring tasks, in stage 1, the pupil diameter of the chatting task is not much different in different takeover scenes, but the pupil diameter of the monitoring task in the obstacle-avoidance scene is larger. However, in NDRTs* takeover scenes, it is hard to draw a conclusion consistent with the single-factor analysis (obstacle-avoidance scene > lane-keeping scene, monitoring > texting), so the relative influence of NDRTs and takeover scenes on pupil diameter cannot be judged.

## 4. Discussions

This research organized shared-driving simulated experiment and sought for drivers’ preferences on NDRTs to decide the NDRTs used in simulated experiment. The different scenes, different TB, different NDRTs, and different takeover stages were compared to explore their influences on workload. This research divided workload into objective workload and subjective workload and sought for factors that have influences on the two kinds of workload, respectively. It revealed how objective and subjective workloads vary under different factors and the interactive influence of factors. The contribution of this research is that factors were combined in pairs to explore their interactive influence on workload, and this is because drivers usually come up with various complex situations, and these situations always include several factors. This research could offer advice for driver workload warning system and promotes traffic safety.

There are still shortcomings in this research. The data in this research are derived from simulated driving, and whether the data analysis conclusions fit with real vehicle driving is not valid. In future research, when the technical conditions, laws, and regulations, etc., are all satisfied, the actual vehicle experimental data [32] will be collected to verify the conclusions. Furthermore, the participants selected in the study are all young people aged 22 to 29. In the future, individual factors, such as age, driving experience, gender, driving style, personality traits, etc., can be considered comprehensively and, combined with different driving environments, explore the safety of takeover in a shared-control environment for more perspective.

## 5. Conclusions

This study is based on the shared driving simulated experiment and uses the driver’s subjective and objective cognitive load data, seeks the subjective and objective cognitive load-influencing factors in the takeover process, reveals the variations of driver’s workload before and after takeover when performing NDRTs, and further explores the interaction mechanism of different NDRTs, TB, etc., on cognitive load and takeover performance.

The study finds that subjective workload has a significant correlation with the difficulty of the takeover scene and TB, a significant negative correlation with NDRT, and a significant positive correlation with subjective distraction score. When in stage 3 and stage 4, TB has a significant correlation with pupil diameter, indicating that the more TB for takeover, the more time the driver will spend to observe the driving environment to ensure the safety of takeover, and this has a high cognitive load. When in stage 2–4, there is a significant correlation with NDRT, indicating that different tasks have an impact on the cognitive load occupied by the takeover.

For subjective workload, it can be concluded that manual operations carried out when drivers think they must take over the vehicle in a timely manner to avoid accident occupy more cognitive resources than when they think it is not a necessary takeover. Different TBs have mere impact on the driver’s subjective workload. When drivers perform relatively more difficult tasks, their subjective workload will increase. Under the influence of the interaction of different factors, when the TB is longer, the total subjective workload score under the same NDRT reduces. In the same takeover scene, the less the TB is, the higher the driver’s workload. When the takeover scene is less dangerous, the subjective workload total score under the same NDRT will decrease.

For objective workload, this study concludes that when the driver has to take over the vehicle to ensure safety under an emergency, it will occupy more workload. When the driver has enough time to take over the vehicle, the brain can provide sufficient cognitive resources for processing driving environment information. For NDRT, monitoring tasks occupy more cognitive load of drivers than texting tasks. The interactive influence of different TBs and different NDRTs on the normalized pupil diameter is consistent with the conclusions of the single-factor study. The effect of TB on the pupil diameter is higher than that of the takeover scene, and the influence of NDRT and takeover scene on the pupil diameter could not be judged.

## Figures and Tables

**Table 1:** Demographic characteristics of participants.

Characteristic Type	Parameter Distribution
Female percentage/%	32.7%
Age/year	24.11 ± 2.23
Driving experience/year	3.68 ± 1.36

**Table 2:** Group design of shared-control driving simulation experiment.

No.	NDRTs (Non-Driving-Related Tasks)	TB (Time Budget) (s)	Takeover Scene
1	Mistake finding	3	Accident ahead
2	Texting	7	Lane line ahead disappears
3	Chatting	7	Lane line ahead disappears
4	Monitoring	3	Accident ahead
5	Chatting	3	Lane line ahead disappears
6	Monitoring	7	Accident ahead
7	Mistake finding	7	Accident ahead
8	Texting	3	Lane line ahead disappears
9	Monitoring	7	Lane line ahead disappears
10	Chatting	3	Accident ahead
11	Texting	3	Accident ahead
12	Mistake finding	7	Lane line ahead disappears
13	Texting	7	Accident ahead
14	Mistake finding	3	Lane line ahead disappears
15	Monitoring	3	Lane line ahead disappears
16	Chatting	7	Accident ahead

**Table 3:** Statistical description of subjective workload total score and distraction degree score.

	Mean	Std Dev	Min	Max	Median
Subjective workload total score	3.82	1.58	1.00	9.40	4.33
Distraction degree score	4.95	2.64	1.00	10.00	5.00
	**Variance**	**Skewness**	**Kurtosis**	**Skewness Z-score**	**Kurtosis Z-score**
Subjective workload total score	2.53	0.17 ± 0.11	−0.03 ± 0.23	1.55	−0.13
distraction degree score	6.98	0.14 ± 0.10	−0.93 ± 0.20	1.41	−4.58

**Table 4:** Start and end time for different stages.

Time Period	Start	End
1	T0 − 3: 3 s before TOR(Take-Over Request)	T0: TOR sent out
2	T0: TOR sent out	T1: drivers takeover
3	T1: drivers takeover	T2: pass by the roadblock
4	T2: pass by the roadblock	T2 + 3: 3 s after passing by the roadblock

**Table 5:** Results of normality test of pupil diameter at different stages.

Stage	Mean	Std Dev	Normal Distribution (Yes/NO)
1	0.491	0.159	Yes
2	0.513	0.141	Yes
3	0.520	0.139	Yes
4	0.529	0.123	Yes

**Table 6:** Correlation analysis between the subjective workload total score and related factors.

		Takeover Scene	TB	NDRT	TB*NDRT	TB*Takeover Scene	NDRT*Takeover Scene	Distracyion Degree Score
Subjective workload total score	Correlation coefficient	−0.213 **	−0.072 *	−0.311 **	−0.153 **	−0.123 **	−0.183 **	−0.544 **
	Significance	0.002	0.049	0.000	0.000	0.000	0.000	0.000

Note: * means *p* < 0.05, ** means *p* < 0.01.

**Table 7:** Correlation analysis between pupil diameter and correlative factors.

Stage		Scene	TB	NDRT
1	Correlation coefficient	−0.047	0.001	0.047
	Significance	0.200	0.979	0.152
2	Correlation coefficient	−0.025	0.050	0.075 *
	Significance	0.500	0.172	0.024
3	Correlation coefficient	−0.027	0.077 *	0.076 *
	Significance	0.460	0.034	0.022
4	Correlation coefficient	0.011	0.125 **	0.100 **
	Significance	0.758	0.001	0.002

Note: * means *p* < 0.05, ** means *p* < 0.01.

**Table 8:** The impact of different takeover scenes on the subjective workload total score.

Takeover Scene	1 Obstacle Avoidance	2 Lane Keeping
Mean	4.075	3.626
Std Dev	1.613	1.482
Mean Rank	274.12	233.96
Standardized TestStatistic	−3.086	
Asymptotic Significance	0.002	

**Table 9:** The impact of different TB on the subjective workload total score.

TB(s)	3	7
Mean	3.997	3.707
Std Dev	1.615	1.503
Mean Rank	266.91	241.45
Standardized TestStatistic	−1.957	
Asymptotic Significance	0.051	

**Table 10:** The impact of different NDRTs on the subjective workload total score.

NDRTs	Mistake Finding	Texting	Chatting
Mean	4.393	3.874	4.017
Std Dev	1.463	1.406	1.554
Mean Rank	304.34	259.864	267.53
Standardized TestStatistic	49.138		
Asymptotic Significance	0.000		

**Table 11:** The influence of different TB*different NDRT on the subjective workload total score.

No.	TB*NDRT	Mean	Sta Dev
1	TB = 3 s*Mistake finding	4.617	1.547
2	TB = 3 s*Texting	4.049	1.418
3	TB = 3 s*Chatting	4.149	1.589
4	TB = 3 s*Monitoring	3.149	1.577
5	TB = 7 s*Mistake finding	4.182	1.356
6	TB = 7 s*Texting	3.699	1.382
7	TB = 7 s*Chatting	3.895	1.524
8	TB = 7 s*Monitoring	2.969	1.518

Note: * means “combine two factors”.

**Table 12:** The influence of different TB*different Takeover scene on the subjective workload total score.

No.	TB*Takeover Scene	Mean	Std Dev
1	TB = 3 s*Obstacle avoidance	4.247	1.698
2	TB = 7 s*Obstacle avoidance	3.907	1.516
3	TB = 3 s*Lane keeping	3.747	1.492
4	TB = 7 s*Lane keeping	3.508	1.469

Note: * means “combine two factors”.

**Table 13:** The influence of different NDRTs*different Takeover scene on the subjective workload total score.

No.	NDRT*Takeover Scene	Mean	Sta Dev
1	Mistake finding*Obstacle avoidance	4.542	1.522
2	Texting*Obstacle avoidance	4.237	1.391
3	Chatting*Obstacle avoidance	4.202	1.709
4	Monitoring*Obstacle avoidance	3.218	1.554
5	Mistake finding*Lane keeping	4.230	1.389
6	Texting*Lane keeping	3.522	1.338
7	Chatting*Lane keeping	3.834	1.375
8	Monitoring*Lane keeping	2.913	1.534

Note: * means “combine two factors”.

**Table 14:** Mean of normalized pupil diameter in different takeover scene and stages.

	Stage 1	Stage 2	Stage 3	Stage 4
Obstacle-avoidance scene	0.497	0.518	0.524	0.529
Lane-keeping scene	0.484	0.506	0.513	0.525

**Table 15:** Mean of normalized pupil diameter in different TB and stages.

	Stage 1	Stage 2	Stage 3	Stage 4
TB = 3 s	0.490	0.505	0.505	0.511
TB = 7 s	0.490	0.519	0.532	0.543

**Table 16:** Mean of normalized pupil diameter in different NDRTs and stages.

	Stage 1	Stage 2	Stage 3	Stage 4
Mistake finding	0.475	0.498	0.510	0.513
Texting	0.473	0.496	0.500	0.509
Chatting	0.523	0.543	0.549	0.552
Monitoring	0.490	0.511	0.515	0.534

**Table 17:** Mean of normalized pupil diameter in different TB, NDRTs, and stages.

	Stage 1	Stage 2	Stage 3	Stage 4
TB = 3 s*Mistake finding	0.480	0.487	0.501	0.499
TB = 3 s*Texting	0.481	0.495	0.497	0.503
TB = 3 s*Chatting	0.506	0.532	0.526	0.527
TB = 3 s*Monitoring	0.493	0.507	0.496	0.517
TB = 7 s*Mistake finding	0.471	0.510	0.519	0.527
TB = 7 s*Texting	0.464	0.497	0.503	0.516
TB = 7 s*Chatting	0.539	0.554	0.571	0.577
TB = 7 s*Monitoring	0.486	0.515	0.535	0.552

Note: * means “combine two factors”.

**Table 18:** Mean of normalized pupil diameter in different TB, takeover scene, and stages.

	Stage 1	Stage 2	Stage 3	Stage 4
TB = 3 s*Obstacle avoidance	0.508	0.529	0.527	0.522
TB = 7 s*Obstacle avoidance	0.485	0.506	0.521	0.536
TB = 3 s*Lane keeping	0.473	0.483	0.485	0.501
TB = 7 s*Lane keeping	0.495	0.531	0.542	0.550

Note: * means “combine two factors”.

**Table 19:** Mean of normalized pupil diameter in different NDRTs, takeover scene, and stages.

	Stage 1	Stage 2	Stage 3	Stage 4
Mistake finding*Obstacle avoidance	0.506	0.522	0.534	0.535
Texting*Obstacle avoidance	0.446	0.477	0.493	0.504
Chatting*Obstacle avoidance	0.519	0.539	0.538	0.532
Monitoring*Obstacle avoidance	0.506	0.519	0.523	0.543
Mistake finding*Lane keeping	0.439	0.470	0.482	0.488
Texting*Lane keeping	0.492	0.509	0.506	0.513
Chatting*Lane keeping	0.526	0.541	0.559	0.574
Monitoring*Lane keeping	0.478	0.505	0.509	0.527

Note: * means “combine two factors”.

## Data Availability

Data sharing not applicable.
